# *OsPOP5*, A Prolyl Oligopeptidase Family Gene from Rice Confers Abiotic Stress Tolerance in *Escherichia coli*

**DOI:** 10.3390/ijms141020204

**Published:** 2013-10-10

**Authors:** Cun-Mei Tan, Rong-Jun Chen, Jian-Hua Zhang, Xiao-Ling Gao, Li-Hua Li, Ping-Rong Wang, Xiao-Jian Deng, Zheng-Jun Xu

**Affiliations:** Rice Institute, Sichuan Agriculture of University, Chengdu 611130, China; E-Mails: tancm058@gmail.com (C.-M.T.); chenrj913@163.com (R.-J.C.); janet.z_87@hotmail.com (J.-H.Z.); myriceworld@hotmail.com (X.-L.G.); lilihua1976@tom.com (L.-H.L.); pingrong_wang@aliyun.com (P.-R.W.); xjdeng2006@aliyun.com (X.-J.D.)

**Keywords:** prolyl oligopeptidase, abiotic stress, protein expression, *E. coli*, *Oryza sativa* L

## Abstract

The prolyl oligopeptidase family, which is a group of serine peptidases, can hydrolyze peptides smaller than 30 residues. The prolyl oligopeptidase family in plants includes four members, which are prolyl oligopeptidase (POP, EC3.4.21.26), dipeptidyl peptidase IV (DPPIV, EC3.4.14.5), oligopeptidase B (OPB, EC3.4.21.83), and acylaminoacyl peptidase (ACPH, EC3.4.19.1). POP is found in human and rat, and plays important roles in multiple biological processes, such as protein secretion, maturation and degradation of peptide hormones, and neuropathies, signal transduction and memory and learning. However, the function of POP is unclear in plants. In order to study POP function in plants, we cloned the cDNA of the *OsPOP5* gene from rice by nested-PCR. Sequence analysis showed that the cDNA encodes a protein of 596 amino acid residues with *M*_w_ ≈ 67.29 kD. In order to analyze the protein function under different abiotic stresses, OsPOP5 was expressed in *Escherichia coli.* OsPOP5 protein enhanced the tolerance of *E. coli* to high salinity, high temperature and simulated drought. The results indicate that *OsPOP5* is a stress-related gene in rice and it may play an important role in plant tolerance to abiotic stress.

## 1. Introduction

Abiotic stress, mainly including drought, salt, and cold, can seriously affect plant growth and development as well as productivity. In order to overcome these adverse stress factors, plants respond and adapt to these adverse circumstance at physiological, biochemical, and molecular levels. Various functional and regulatory proteins play an important role in controlling abiotic stress tolerance [1]. Some studies have shown that plant proteases play a significant role in storage protein mobilization during defense system activity [2,3] and senescence [4]. However, the enzymatic properties and physiological functions of a great number of these proteases remain unknown.

Prolyl oligopeptidase (POP, also known as prolyl endopeptidase) was first discovered in 1979. The prolyl oligopeptidase family of serine proteases (clan SC, family S9) includes four members in plants, these members are prolyl oligopeptidase (POP, EC3.4.21.26), dipeptidyl peptidase IV (DPPIV, EC3.4.14.5), oligopeptidase B (OPB, EC3.4.21.83), and acylaminoacyl peptidase (ACPH, EC3.4.19.1), and they have been identified and their enzymatic properties have been characterized [5–8]. The serine type of endopeptidase shows activity in the cytosol, but is still not able to digest polypeptides containing more than 30 amino acid residues. POP cleaves the bound peptide after proline residues, and at a much slower reaction rate at Ala residues; such specificity sharply contrasts with other serine proteases [9,10]. So far, several complementary DNAs (cDNAs) of the prolyl oligopeptidase family related proteins have been cloned from many organisms, and some of them lacking the catalytic serine residues were inactive [11,12]. Studies of the structural relation between these enzymes and lipases [13], as well as their secondary structure [14], indicates that these enzymes are considered as members of the α/β-hydrolase fold enzymes.

The homology of amino acid sequence between the four basic peptidases is quite low, but recent crystal structure determination and modeling analysis suggests that they have a similar three-dimensional (3D) structure, and the crystal structure showed that POP contains a catalytic (peptidase) domain with an α/β hydrolase fold and a 7-bladed β-propeller domain [6]. The β-propeller domain contains the active site that is situated in a central cavity at the domain interface [15]. Polgár [6] reported that apart from the regulatory effect, the propeller domain, originally independent of the catalytic domain, also offers a residue that significantly modifies the catalytic action of the peptidase domain. Therefore, prolyl oligopeptidase and oligopeptidase B are in the branch of endopeptidase and are found in the cytoplasm. In addition to acylaminoacyl peptidase and dipeptidyl peptidase IV, which are exopeptidases, acylaminoacyl peptidase is a cytoplasmic omega peptidase; in addition, dipeptidyl-peptidase IV is a membrane-binding enzyme that can decompose dipeptides from the amino terminus of oligopeptide [9].

*POP* was found in human as an oxytocin-degrading enzyme [16,17] and is present in most tissues and organisms. *POP* has been cloned from different organisms including porcine brain [18], human lymphocytes [19], mouse brain [20], and bovine brain [21]. The structure and localization of mouse prolyl oligopeptidase genes were also characterized [22]. Prolyl oligopeptidase may exhibit the highest concentration among brain peptidases compared to other peptidase, and thus it is implicated in a variety of disorders of the central nervous system. Furthermore, Oligopeptidase B was first isolated from *E. coli* cells with thadpsin-like specificity and the ability to decompose peptides at lysine and arginine residues [23], but its role in *E. coli* is unknown. The physiological role of this enzyme is not clear, and its physiological substrate has not identified. Until now, *OPB* was only discovered in prokaryotes and unicellar eukaryotes. In fact, trypanosome *POP* and *OPB* both seem to be important virulence factors [24]. Deletion of the *OPB* gene results in a marked decrease in trypomastigote virulence and capacity for cell invasion [25].

Different from both prolyl oligopeptidase and oligopeptidase B, Dipeptidyl-peptidase IV is a dimer, an exopeptidase, a glycoprotein and an ectoenzyme bound to the cell membrane. It cleaves dipeptidase at the penultimate proline or to a lesser extent at alanine from the amino terminus of the oligopeptide [26,27]; its cellular localization and enzymatic properties also vary from other dipeptidyl-peptidases. In contrast to prolyl oligopeptidase and oligopeptidase B, acylaminoacyl peptidase is a monomer [9].

In this research, we cloned a rice *POP* gene and investigated its expression pattern under abiotic stress in rice. To further analyze the characteristics of *OsPOP5*, we introduced *OsPOP5* into the pET32a vector expressed in *E. coli*. Expression of *OsPOP5* enhanced the tolerance of *E. coli* to dehydration, heat, and high salinity, which suggested that *OsPOP5* may play a protective role under stressed conditions.

## 2. Results and Discussion

### 2.1. Results

#### 2.1.1. Cloning and Sequence Analysis of *OsPOP5* Gene

**Figure 1:**
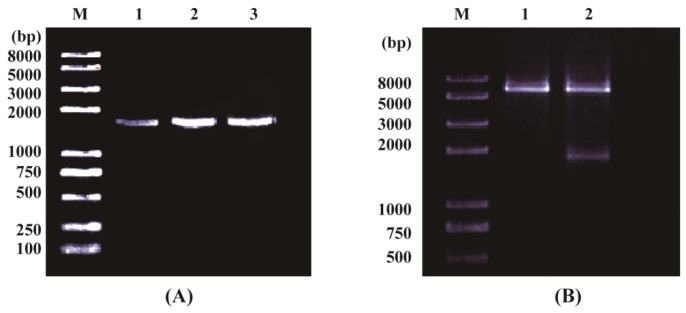
Polymerase chain reaction (PCR) amplification and restriction analysis of *OsPOP5* in rice. (**A**) OsPOP5 was isolated and amplified as described in Materials and Methods Section. Lanes **1**, **2** and **3** are all *OsPOP5* gene amplified in rice. Lane **M**: DNA ladder (PCR marker); Lanes **1**, **2** and **3**, PCR product of OsPOP5 (~1804 bp); (**B**) Restriction analysis of pET32a-OsPOP5. Lane **M**: DNA ladder (PCR marker); Lane **1**: restriction analysis of pET32a; Lane **2**: restriction analysis of pET32a–OsPOP5 (~1790 bp).

The *OsPOP5* gene was cloned from cDNA library of rice (*Oryza sativa*) by nested polymerase chain reaction (PCR) and sequenced (Figure 1). Sequence analysis indicated that the complete open reading frame of *OsPOP5* cDNA is 1791 bp. The predicted protein of OsPOP5 comprises 596 amino acids with the calculated molecular mass of 67,294.7 Da and the isoelectric point (PI) of 6.00. The *OsPOP5* cDNA sequence has been submitted to GenBank with accession NM_001053127. InterProscan and MOTIF search results suggested that it has a DPPIV_N (Dipeptidyl Peptidase IV *N*-terminal region) and Peptidase S9 (prolyl oligopeptidase family) domain, and it might belongs to the prolyl oligopeptidase family.

**Figure 2:**
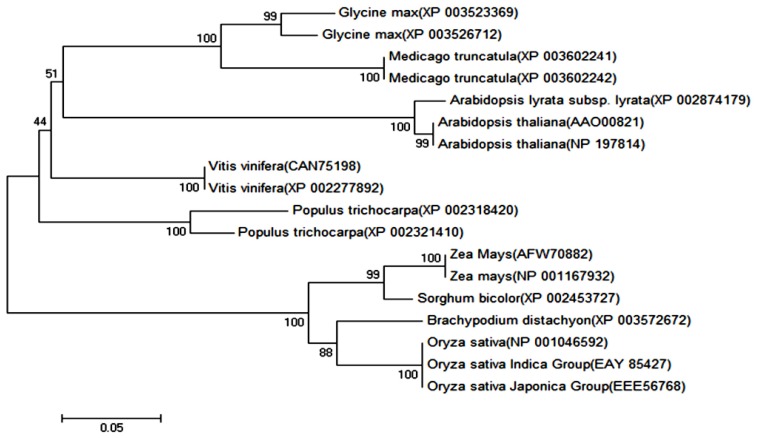
The phylogenetic analysis of prolyl oligopeptidase from rice and other plant species by MEGA 5.05 from CLUSTAL W alignments. The neighbor-joining method was used to construct the tree with p-distance. The number for each interior branch is percent bootstrap value (100 replicates).

To investigate the evolutionary relationship of OsPOP5 proteins in plants, a phylogenetic tree was constructed using Neighbor-Joining method with the full-length amino acid sequences. We used the full-length amino acid sequences with BLAST (basic local alignment search tool) [28] and ClustalW [29], conducting multiple sequence analysis. BLASTP (protein blast) [28], analysis showed OsPOP5 has 100% amino acid similarity to *Oryza sativa* indica Group (Accession No. EAY_85427.1), 100% to *Oryza sativa* Japonica Group (Accession No. EEE_56768.1), 90% to *Brachypodium distachyon* (Accession No. XP_003572672.1), 89% to *Sorghum bicolor* (Accession No.XP_002453727.1), 88% to *Zea Mays* (Accession No. NP_001167932.1), 87% to *Zea Mays* (Accession No. AFW70882.1). Phylogenetic tree result indicated that *OsPOP5* was clustered with *Oryza sativa* indica Group, *Oryza sativa* Japonica Group, *Zea Mays*, *Sorghum bicolor*, *Brachypodium distachyon*, whereas other POP proteins were categorized into a different large branch (Figure 2).

**Figure 3:**
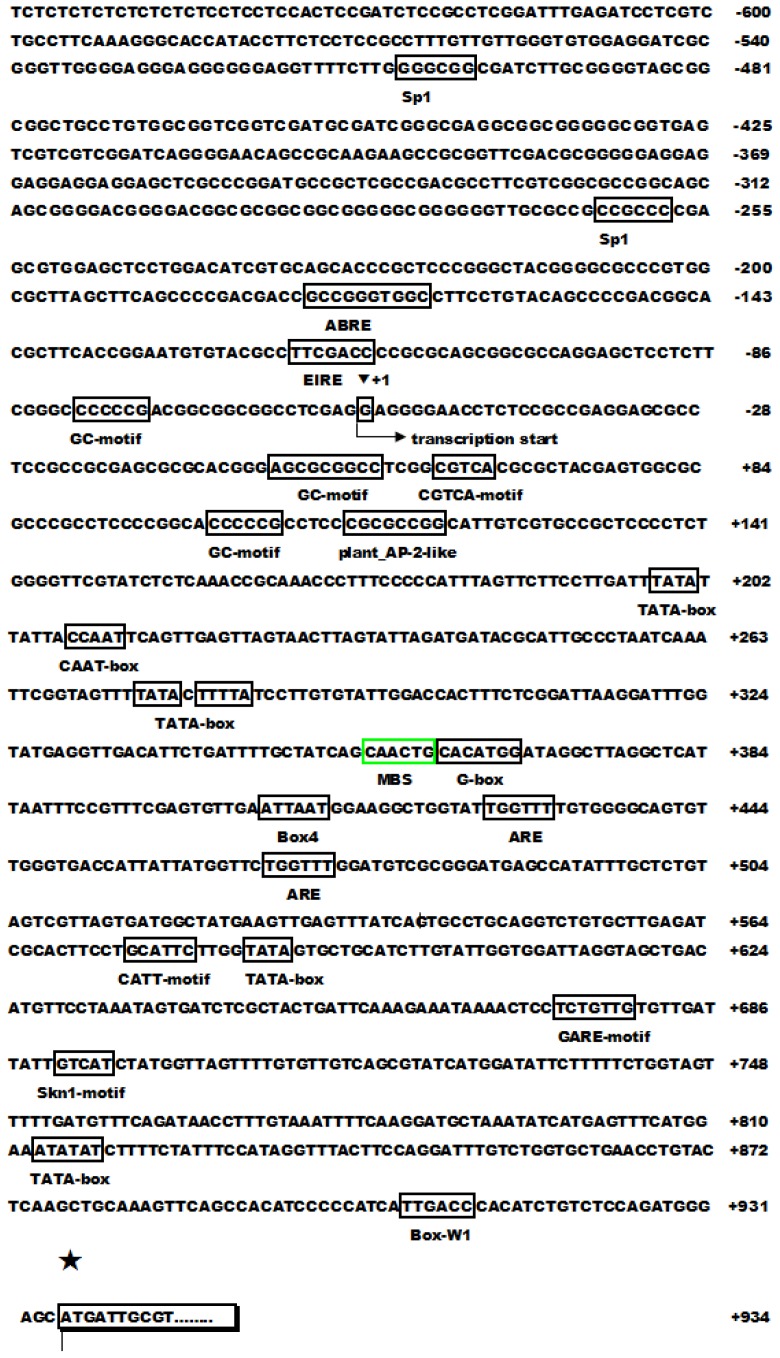
Promoter analysis of *OsPOP5*. The putative *cis*-acting elements are boxed, and translation starts sites are indicated with arrows. Black inverted triangle (+1): transcription start. Black star: translation start.

We then analyzed the upstream *cis*-acting regulatory element of *OsPOP5* and found that stress and defense related elements (Figure 3), such as W-box, Box-W1 (fungal elicitor responsive element), Sp1 (light responsive element), TCA-element (*cis*-acting element involved in salicylic acid responsiveness), and ABRE (*cis*-acting element involved in the abscisic acid responsiveness) were enriched in the promoter region of *OsPOP5*, indicating that these stress related *cis*-acting elements may be responsive for stress regulated expression of *OsPOP5*.

#### 2.1.2. Expression of *OsPOP5* under Different Stress Treatments

**Figure 4:**
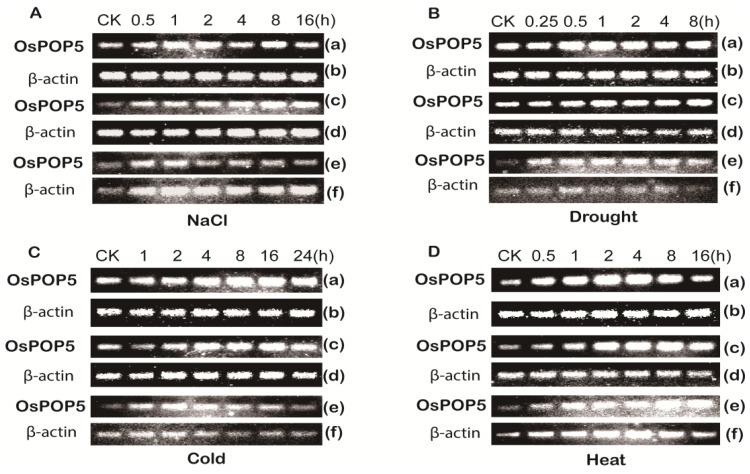

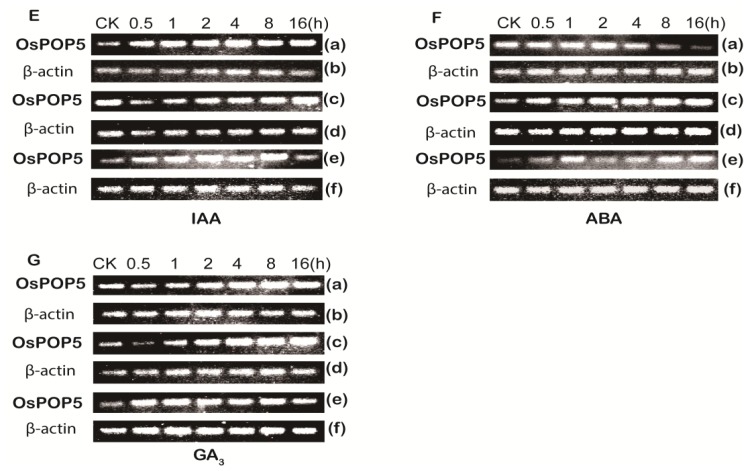
Expression analysis of *OsPOP5* under various abiotic stress treatments in different tissues. The expression analyses were performed by total RNA extracted from leaves (**a** and **b**), stems (**c** and **d**) and roots (**e** and **f**) of rice at marked time after treatment with (**A**) NaCl; (**B**) drought; (**C**) cold; (**D**) heat; (**E**) indole-3-acetic acid (IAA); (**F**) abscisic acid (ABA); and (**G**) gibberellin (GA_3_), respectively. The β-actin was used as standard control to show the normalization of amount of template PCR reactions.

To determine whether *OsPOP5* is involved in abiotic stress response, RT-PCR assay was performed to analyze *OsPOP5* expression in rice under drought, cold, heat, salt stress and chemical treatment including abscisic acid (ABA), indole-3-acetic acid (IAA) and gibberellin (GA_3_) in different tissues. The result showed that *OsPOP5* was ubiquitously expressed in rice root, stem, and leaf (Figure 4). In salt treatment, the expression level of *OsPOP5* was upregulated after 0.5 h and remained high throughout the treatment in leaf and stem. However, in root, the expression level was up-regulated after 0.5 h and down-regulated at 2 h and maintained this level. In response to drought, the *OsPOP5* transcript showed no significant change in leaf and stem, and the transcript was upregulated in root after 0.25 h treatment, meanwhile, the *OsPOP5* expression level was downregulated in the control. In cold and heat stress treatment, the *OsPOP5* showed similar transcript levels in leaf and stem. However, the *OsPOP5* expression level was upregulated in 1 h and downregulated in 24 h in cold stress in root. In heat stress, the *OsPOP5* transcript was significantly increased in 1 h and reached its peak at 16 h in root. We also analyzed *OsPOP5* response to plant hormones including IAA, ABA and GA_3_. Under IAA treatment, the *OsPOP5* transcript level increased after 0.5 h in leaf, which decreased after 0.5 h in stem and increased after 1 h. In response to ABA treatment, the *OsPOP5* transcript decreased at 8 h, and there was no obvious change in stem and root. The GA_3_ treatment result was similar with IAA. All of these results indicate that the *OsPOP5* gene is responsive to all stresses tissue specifically.

#### 2.1.3. Expression Analysis of OsPOP5 in *E. coli* by SDS-PAGE

**Figure 5:**
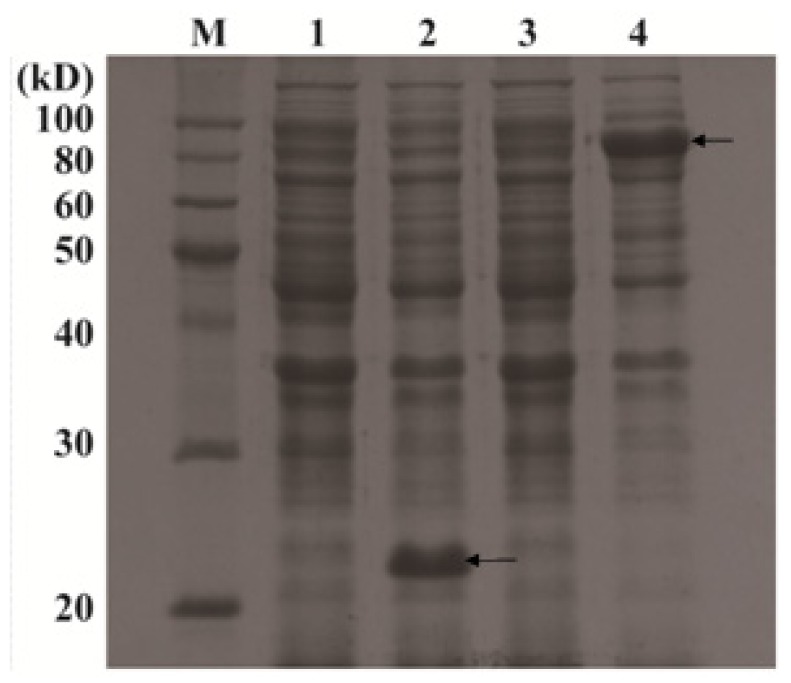
SDS-PAGE analysis of *OsPOP5* overexpression in *E. coli* BL21. **M** marker (Protein Ruler II); lane **1** untreated BL/pET32a; lane **2** BL/pET32a induced by isopropylthio-β-d-galactoside (IPTG) (arrow mark is 21 kD); lane **3** untreated BL/OsPOP5; lane **4** BL/OsPOP5 induced by IPTG (arrow mark is 88 kD). Total proteins of BL/pET32a and BL/OsPOP5 from supernatants were separated by SDS-PAGE. The thicker bands near the calculated molecular mass of TrxA intein and TrxA-OsPOP5 fusion proteins were about 21 and 88 kDa, respectively.

The recombinant plasmid pET32a–OsPOP5 and empty vector pET32a as a control were transformed into *E. coli* BL21. The recombinant protein was induced after 2 h of isopropylthio-β-d-galactoside (IPTG) treatment and reached maximum levels at 6 h as confirmed by SDS-PAGE. The supernatants of treated cell lysates from the IPTG-induced transformed cells were studied. According to the construction of recombinant plasmids, the size of the proteins deduced from BL/pET32a and BL/OsPOP5 cells should be about 21 and 88 kDa, respectively. Figure 5 shows a specific band about 21 kDa in BL/pET32a cells and another thicker band of the 88 kDa fusion protein was obviously observed on gels containing the BL/OsPOP5 cell supenatant (Figure 5). These results indicated that the OsPOP5 fusion protein was successfully expressed.

#### 2.1.4. Overexpression of *OsPOP5* in *E. coli* Enhances Growth during Abiotic Stresses

**Figure 6:**
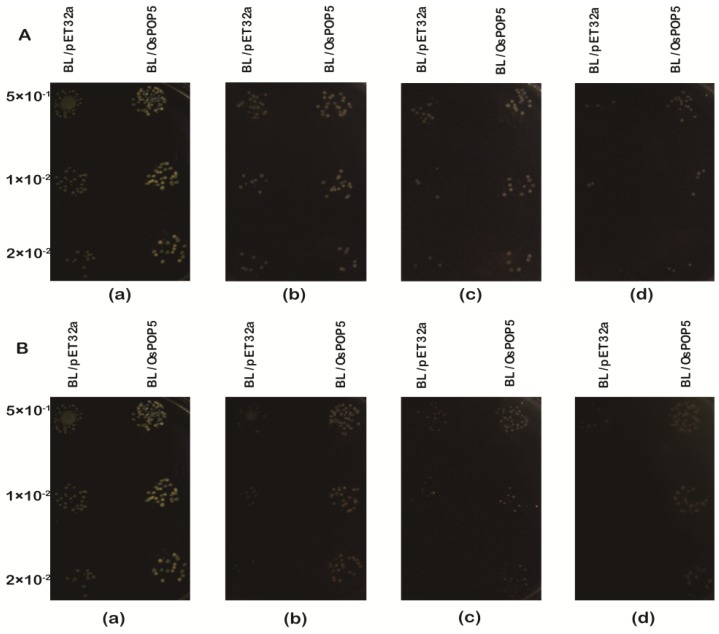

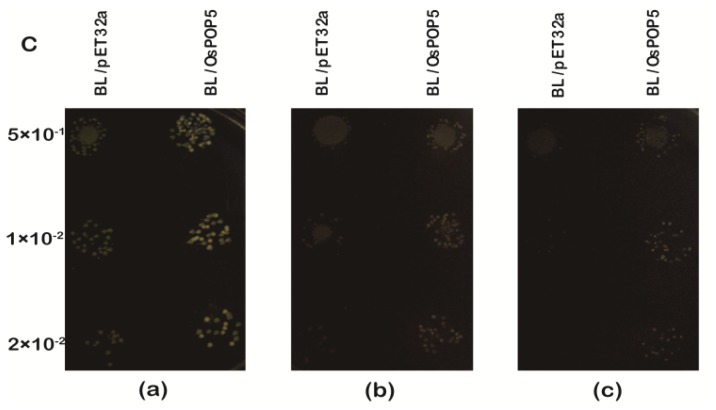
The growth performance of BL/pET32a and BL/OsPOP5 cells. (**A**) The *E. coli* cells were subjected to 50 ºC for 0.5 to 2 h, and then 10 mL from 5 × 10^−1^ to 2 × 10^−2^ dilutions were spotted onto Luria-Bertani (LB) plates containing 1 mmol/L IPTG. (**a**) Control (LB plate); (**b**) 50 ºC 0.5 h; (**c**) 50 ºC 1 h; (**d**) 50 ºC 2 h; (**B**) Spot assay of BL/pET32a and BL/OsPOP5 on the LB plates with different concentration of NaCl (**a**) control (LB plate); (**b**) 400 mM NaCl; (**c**) 500 mM NaCl; (**d**) 600 mM NaCl; (**C**) Spot assay of BL/pET32a and BL/OsPOP5 on the LB plates with different concentration of mannitol (**a**) control (LB plate); (**b**) 500 mM mannitol; (**c**) 800 mM mannitol. IPTG was added to the cultures of BL/pET32a and BL/OsPOP5 to induce the expression of recombinant protein. The cultures were adjusted to OD600 = 0.5–0.6 and final concentration up to 1 mmol/L. Ten microliters from 5 × 10^−1^ to 2 × 10^−2^ dilutions were spotted onto LB plates.

To examine the effect of the overexpression of the OsPOP5 protein on the growth of *E. coli* recombinants under different environment stresses, cultures of BL/pET32a and BL/OsPOP5 were diluted and spread on different plates. Figure 6 showed that recombinant and control cells have similar growth on Luria-Bertani (LB) medium in overnight grown culture, whereas, in high salinity, drought and high temperature supplemented medium, the recombinant cells increased the number of colonies as compared to control cells.

Figure 6 shows that the number of colonies varied on plates supplemented with 400 to 600 mM NaCl, and that the viability of BL/OsPOP5 cells were significantly greater than BL/pET32a cells under various NaCl concentrations. Moreover, the cell size of BL/OsPOP5 in the medium supplemented with 400 mM NaCl plates was bigger than that of BL/pET32a on 500 and 600 mM NaCl. When NaCl concentration was increased 600 mM in the mediums, there were small and few BL/OsPOP5 cells found *versus* nothing in the control. In addition, similar results were found with mannitol treatment; the colony number of BL/OsPOP5 was much higher than that of BL/pET32a at high salt and high mannitol concentrations. These results indicate that the expression of the *OsPOP5* gene increased the salt and drought tolerance of *E. coli* cells.

In order to identify the effect of overexpression of *OsPOP5* protein on the growth of *E. coli* recombinants under high-temperature stresses, cultures with IPTG were transferred to 50 ºC. After the temperature treatment for different time periods, the number of cells was calculated in diluted culture aliquots. Although the number of cells reduced rapidly in both cultures upon heat shock, BL/*OsPOP5* cells were numerous compared with control cells after high temperature treatment and about 2-fold after 1 h at 50 ºC. Most of the BL/pET32a cells died after 1.5 h at 50 ºC, whereas fewer BL/OsPOP5 cells died under the same condition. These results revealed that the *OsPOP5* gene significantly induced tolerance to high-temperature stress. Moreover, we investigated the tolerance of BL/OsPOP5 and BL/pET32a on plates with different concentrations of mannitol. The results showed BL/OsPOP5 and BL/pET32a exhibited almost no difference at 500 mM, but a significant difference was found at 1M. This result indicated that the *OsPOP5* gene enhanced tolerance to dehydration stress.

### 2.2. Discussion

A great number of genes in plants are induced after exposure to various abiotic stresses: they encode receptors, kinases, transcription factors, and other signalling molecules. These genes help enhance stress tolerance in plants in various ways [30,31]. The sequence of various *cis*-acting elements, including ABRE, G-boxes, GCC-boxes, MBSs, TGA-motif, TGAGC-motif and motif IIb, are present in the 1.5 kb promoter region of *OsPOP5* (Figure 1B). The ABRE and MBS are known as recognition sites of the ABRE/ABF transcription factor and the MYB biding site involved in drought response, respectively [32]. These *cis*-elements and their respective transcription factors have important roles in ABA signaling and abiotic stress responses [33]. The W-box and GCC boxes are known as recognition sites for WRKY and ERF transcription factors, respectively [34,35]. These appear to be involved in the regulation of various plant-specific physiological processes such as pathogen defense and senescence. The TCA-element, TGA-element, TGACG-motif and motif IIb are known as *cis*-acting elements involved in salicylic acid responsiveness, auxin responsive elements (AuxREs), *cis*-acting element involved in MeJA responsiveness and abscisic acid responsive elements, respectively. These sequences may function as abiotic and/or biotic stress-responsive *cis*-acting elements in the *OsPOP5* promoter.

ABA plays important roles in adapting vegetative tissues to abiotic stresses such as drought and high salinity, as well as in seed maturation and dormancy. ABA regulates the expression of many genes that might function in dehydration tolerance in both vegetative tissues and seeds [36,37]. The *OsPOP5* gene showed no significant induction by various stresses and chemical treatments in RT-PCR (Figure 3). From the promoter analysis we found that the *OsPOP5* was a stress related gene, but it was not significantly changed by RT-PCR analysis. There is an ABRE *cis*-element in the OsPOP5 promoter, and ABRE is a major *cis*-element in ABA-responsive gene expression. Moreover, a single copy of ABRE is not sufficient for ABA-responsive transcription [32]. ABRE and coupling elements such as CE1 and CE3 constitute an ABA-responsive complex in the regulation of wheat *HVA1* and *HVA22* genes. Two ABRE sequences are necessary for expressing Arabidopsis *RD29B* in seeds and for the ABA-responsive expression of *RD29B* in vegetative tissue. One of these ABRE sequences might function as a coupling element. Most of the known coupling elements are similar to ABREs and contain an A/GCGT motif. Either an additional copy of ABRE or coupling elements are necessary for ABA-responsive gene expression.

POP may play a role in many biological processes, such as maturation and degradation of peptide hormones and neuropathies [38,39], memory and learning [26], signal transduction [40] and protein secretion [41]. Recently, POP was reported to have roles associated with cell cycle regulation [42], angiogenesis [43] and cellular signaling [44]. In addition, there appears to be some consensus that POP possesses a non-hydrolase function and it was demonstrated that POP is located in close association with the cytoskeletal component tubulin [41]. POP has been considered as a housekeeping enzyme, limited to the cytoplasm [6], and experimental data has shown that that expression is neither exclusively cytoplasmic [45,46] nor homogeneous across the tissues [47].

In human and rat, POP enzyme activity has been obtained in different tissues [48], and high activities have been measured in the brain. In the brain, the distribution of immune-reactive POP protein [47] was partly separated from the locations of enzyme activities or POP mRNA levels [49], pointing to endogenous and post-translational regulation of POP. Brain POP has been found also in the membrane and POP protein has also been detected in nuclei. Furthermore, prolyl oligopeptidase enzyme activities have been previously measured in some peripheral tissues such as skeletal muscle, testis, liver, kidney, lung, renal cortex and gut [45]. However, the physiological function of POP and its enzyme activity in plants has remained unclear.

Taken together, owing to our results, it is reasonable to speculate that the protective mechanism of *OsPOP5* might be similar in prokaryotes and eukaryotes under environmental stress conditions, and it is expected that heat, salt, drought, and other stress tolerant crops will be developed through overexpression of *OsPOP5* from rice. Furthermore, to clarify the mechanism of such stress tolerance in plants, it will be important in the future to determine exactly which proteins interact with OsPOP5.

## 3. Experimental Section

### 3.1. Plant Material and Abiotic Treatments

The seeds of rice (*Oryza sativa L. Sub. japonica cv. Nipponbare)* were sterilized in 70% ethanol and 1% NaClO, and germinated at 30 ºC in the growth chamber. All stress and chemical treatments were applied to 2-week-old seedlings. Dehydration was induced by placing plants on a dry filter paper in air. Cold and heat treatments were applied by transferring plants to a growth chamber set to 4 and 42 ºC, respectively. Salinity treatments were applied by submerging the roots of plants in 250 mM NaCl solution. For chemical treatments, the roots of plants were submerged in an aqueous of abscisic acid (ABA, 10 mg/L), gibberellin (GA_3_, 5 μM), indole-3-acetic acid (IAA, 20 μM), respectively. The leaves, stems and roots were collected at 0, 0.5 (or 1), 2, 4, 8, 16, 24 and 48 h for each treatment. All tissue samples were immediately frozen in liquid nitrogen and stored at −70 ºC until use.

### 3.2. RNA Isolation and Reverse Transcription (RT)

Total RNA was extracted from various stress-treated seedlings of rice using Trizol reagent (Invitrogen,, Shanghai, China) according to the manufacturer’s protocol. The RNA was subsequently treated with RNase-free DNase I (Takara, Dalian, China) to remove the remaining genomic DNA. The first strand cDNA was synthesized using TransScript™ First-strand cDNA Synthesis SuperMix (TransGen, Beijing, China). The harvested complementary cDNA was stored at −20 ºC until use.

### 3.3. Isolation and Sequence Analysis of *OsPOP5*

The full-length cDNA of *OsPOP5* was obtained using gene-specific primers by nested PCR. The gene-specific primers described as follows: P1: 5′-TCTGGTGC-TGAACCTGTACTCAAG-3′, P2: 5′-CATACTGTAACAACATCCCAAGTCAT-3′, P3: 5′-ATGATTGCGTATGTGCGGGATGATG-3′, P4: 5′-TCCTCTCTTCACATTCACAGGTTCC-3′. The PCR products were cloned into the pEASY-Blunt cloning vector (TransGen, Beijing, China) and subjected to sequencing.

Homology searches were performed using blast with default parameters at NCBI [28]. Multiple sequence alignment of prolyl oligopeptidase protein was performed using ClustalW [29]. The phylogentic tree was performed using MEGA 5.05 program by neighbor-joining (NJ) method.

### 3.4. Semi-Quantitative PCR Analyse

The primers for *OsPOP5* were 5′-GAACTGGTTATGGTCAAGGCTG-3′ and 5′-ACACGGAGC GTTGTAGAAG-3′ and those for β-actin were 5′-TTCAGCAGCTGTCTCCAGAGAT-3′ and 5′-CTCGATTGTCCATCTTCCAAACT-3′. The PCR program includes an initial denaturation at 94 ºC for 5 min, 25 cycles at 94 ºC for 30 s, 62 ºC for 30 s, 72 ºC for 30 s, and a final 72 ºC for 10 min. In addition, a 250-bp PCR fragment of the rice constitutively expressed actin gene was amplified as an internal control to ensure equal amount of cDNA used in each RT-PCR reaction.

### 3.5. Construction of *OsPOP5* Expression Vector

In order to construct the expression vector pET32a-OsPOP5, Primers with single restriction enzyme site for *Eco*RI were designed: 5′-GATCCGAATTC-ATGATTGCGTATGTGCGGGAT-3′, 5′-AGCTCGAATTCCAGGTTCCTCTCTACAAAATC-3′. The pET32a vector using *Eco*RI digestion and apply by Alkaline CIAP (Takara, Dalian, China) dephosphorylation. The amplified products were cloned into pET32a vector at *Eco*RI site to express OsPOP5 protein fused with Trx Tag™ thioredoxin at the *N*-terminus. The pET32a–OsPOP5 plasmid was transformed into *E. coli* BL21 (DE3). The BL21 cell with pET32a–OsPOP5 plasmid was named as BL/OsPOP5. The transformant of pET32a was used as a control and was named BL/pET32a.

### 3.6. OsPOP5 Expression in *E. coli* BL21

Expression of OsPOP5 in *E. coli* BL21 was carried out as follows: Both BL/OsPOP5 and BL/pET32a were grown at 37 ºC in Luria-Bertani (LB) agar medium supplemented with ampicillin (50 μg/mL). After 3–4 h growth until OD_600_ reached 0.5–0.6, isopropylthio-β-d-galactoside (IPTG) was added to cultures to a final concentration of 1.0 mM and grown at 37 ºC for 3–4 h to induce expression of the inserted gene in recombinants. The cell culture pellet was collected by centrifugation at 13,000× *g* for 5 min and resuspended in 300 uL PBS buffer (137 mM NaCl, 2.7 mM KCl, 10 mM Na_2_HPO_4_, 2 mM KH_2_PO_4_). Twenty microliters samples with added 2 μL 5× SDS-PAGE sample loading buffer [250 mM Tris-HCl (pH 6.8), 10% (*w*/*v*) SDS, 0.5% (*w*/*v*) BPB, 50% (*v*/*v*) glycerin, 5% (*v*/*v*) β-Mercaptoethanol] were placed in eppendorf tubes and boiled for 8 min. The samples were fractionated on 12% SDS-PAGE, and finally gels were stained with Coomassie brilliant blue.

### 3.7. Assays for Abiotic Stress Tolerance of *E. coli* Transformants

Stress tolerance assays of BL/OsPOP5 and BL/pET32a were analyzed. IPTG induction and cell cultures were prepared as described above. The concentration of all induced cultures in liquid LB was adjusted to OD_600_ value of 1.0. To measure salt tolerance of transformed *E. coli* cells, the samples were diluted by 50-fold, 100-fold, 200-fold and then 10 μL of the diluted samples was spotted on IPTG LB agar plates containing 50 μg/mL ampicillin and 1.0 mM IPTG and supplemented with additional 400, 500, and 600 mM concentration gradient of NaCl, 500 mM, 800 mM and 1 M mannitol for drought treatment, respectively. For the assay of the thermophylactic experiments, 1 mL samples were transferred to 50 ºC. Aliquots (100 μL) were taken at different periods 0.5, 1, 1.5, 2, 2.5, and 3 h successively, and then 10 μL of samples diluted by 50-fold, 100-fold and 200-fold was spread onto IPTG LB agar plates. For the assay of cryophylactic experiments, the samples were kept frozen for 24 h at −80 ºC and allowed to thaw at ambient temperature (32 ºC) for 1 h, which constituted one freeze—thaw cycle. Aliquots (100 μL) were taken after 2, 4, 6, 8, 10, and 12 cycles successively, and then 10 μL of dilutions (1:10) was spread onto IPTG LB agar plates.

## 4. Conclusions

In this study, the gene encoding a prolyl oligopeptidase family gene, *OsPOP5* was cloned and characterized in rice for the first time. Analysis of the gene structure shows that OsPOP5 encodes a 596 amino acid protein with a PI of 6.00. There are ABRE, G-boxes, GCC-boxes, MBSs, TGA-motif, TGAGC-motif and motif IIb *cis*-elements in the *OsPOP5* promoter. Semi-quantitative RT-PCR analysis results indicate *OsPOP5* is a stress related gene. Heterologous expression of OsPOP5 protein in *E. coli* enhanced *E. coli* abiotic stress tolerance, and thus implies that *OsPOP5* may plays an important role in plant tolerance to abiotic stress.
